# Predicting Posttraumatic Stress Disorder Risk: A Machine Learning Approach

**DOI:** 10.2196/13946

**Published:** 2019-07-22

**Authors:** Safwan Wshah, Christian Skalka, Matthew Price

**Affiliations:** 1 University of Vermont Burlington, VT United States

**Keywords:** PTSD, machine learning, predictive algorithms

## Abstract

**Background:**

A majority of adults in the United States are exposed to a potentially traumatic event but only a handful go on to develop impairing mental health conditions such as posttraumatic stress disorder (PTSD).

**Objective:**

Identifying those at elevated risk shortly after trauma exposure is a clinical challenge. The aim of this study was to develop computational methods to more effectively identify at-risk patients and, thereby, support better early interventions.

**Methods:**

We proposed machine learning (ML) induction of models to automatically predict elevated PTSD symptoms in patients 1 month after a trauma, using self-reported symptoms from data collected via smartphones.

**Results:**

We show that an ensemble model accurately predicts elevated PTSD symptoms, with an area under the curve (AUC) of .85, using a bag of support vector machines, naive Bayes, logistic regression, and random forest algorithms. Furthermore, we show that only 7 self-reported items (features) are needed to obtain this AUC. Most importantly, we show that accurate predictions can be made 10 to 20 days posttrauma.

**Conclusions:**

These results suggest that simple smartphone-based patient surveys, coupled with automated analysis using ML-trained models, can identify those at risk for developing elevated PTSD symptoms and thus target them for early intervention.

## Introduction

### Background

Posttraumatic stress disorder (PTSD) is a psychiatric condition that leads to significant disability and impairment [1]. Early interventions administered shortly after a traumatic event can reduce the onset of PTSD and associated long-term impairment [2]. Given the costs associated with early intervention, it is not feasible or necessary to intervene with everyone exposed to these events—rather, a screen-and-treat approach is recommended in which those at high risk for PTSD are identified and treated. A key barrier to providing early intervention is an inability to accurately identify those at high risk for PTSD in this acute posttrauma period (<30 days following an event). The limited ability to detect those at risk stems from a limited understanding of how PTSD symptoms develop and, thus, what factors are most helpful in determining risk for the disorder.

A diagnosis of PTSD requires symptoms to be present for at least 30 days. Previous studies suggest that symptoms first appear in the days and weeks after a traumatic event and gradually increase over time [3,4]. Therefore, it may be possible to identify those at risk for PTSD by monitoring the progression of symptoms during this early period. Other previous studies have shown that effective monitoring and data collection can be implemented via smartphone surveys [5,6]. We hypothesized that predictive models based on statistical correlations between observable symptoms shortly after a traumatic event and eventual PTSD symptomology can be developed. Such predictive models would allow individuals at elevated risk for more severe psychopathology to be identified and provided with an early intervention.

In this paper, we take the initial steps toward such a predictive model. We investigated whether correlations exist between PTSD symptoms present shortly after trauma and at 1 month after an event and whether these correlations can be discovered by supervised *machine learning* (ML) approaches. Previous studies have shown that ML techniques are effective for predictive modeling in a medical setting, for example, to predict cancer prognoses [7]. However, such models have yet to be regularly implemented in psychiatric conditions. Furthermore, models induced by ML can be thoroughly vetted by techniques such as cross-validation, increasing confidence in their relevance.

The study presented here uses data collected during a clinical study involving 90 individuals who experienced a criterion A traumatic event and who were recruited from the critical care service of a level-1 trauma center in Northern New England [6]. PTSD symptoms were assessed using validated clinical scales. For this study, we prepared disjoint training, testing, and cross-validation datasets from the provided data for ML analysis. Our dataset is described in further detail in the Dataset section.

In this study, we took a comparative approach to investigating not only whether predictive ML-induced models may exist but also which are the best approaches to model the induction. The development of PTSD symptoms among those who go on to have severe PTSD symptoms follows a complex course, which may be nonlinear [3,8]. Hence, we considered nonlinear ML techniques, in particular, support vector machines (SVMs) with nonlinear kernels and random forest (RF). We also emphasized ensemble techniques that combine predictions from multiple models to obtain an improved prediction.

Specifically, this study considered 4 research hypotheses:

- Hypothesis 1: ML can demonstrate significant statistical correlations between observable symptoms and elevated PTSD 1 month after trauma.
- Hypothesis 2: ML can identify the relevance of early symptoms used to predict PTSD by care providers.
- Hypothesis 3: ML can identify the number of days needed to predict elevated PTSD 1 month after trauma.
- Hypothesis 4: ML-induced models can be used to predict elevated PTSD 1 month after a trauma, given that symptoms are displayed between 10 and 20 days posttrauma.

### Dataset

In this section, we describe the dataset we used for our study, which was collected during a clinical study involving 90 individuals who experienced a criterion A traumatic event and were recruited from the critical care service of a level-1 trauma center in Northern New England [6]. We also describe our data preprocessing methods and feature correlation and feature importance analyses on the preprocessed data.

#### Data Collection

To recruit participants in the cited study [6], a trained research assistant approached the prospective participants at the bedside in the hospital and administered an initial assessment battery to determine if the trauma they experienced met the criterion A for a diagnosis of PTSD. Participants were met bedside by a care provider within a mean of 4.88 days and an SD of 5.22 days after their traumatic event. Participants then downloaded a mobile app to their device that administered the assessment surveys. The app used for this study was Metricwire [9], a platform that allows the administration of self-reported surveys on a mobile device over a predefined period. Metricwire was available for download for free from the respective app stores.

Participants (N=90) were aged mean 35 (SD 10.41) years, were a majority of males (n=57), and had completed college (n=36). The sample was predominately white (n=80). The most common type of injury was motor vehicle accident (n=45). Cell phone ownership included 52 iPhones and 35 Android devices. In addition, 3 participants identified having another type of device but had access to an Android or iPhone device. PTSD symptoms were assessed with the PTSD checklist-5 at 1-month posttrauma [10]. According to the Diagnostic and Statistics Manual 5^th^ Edition (DSM-5) criteria, the PTSD checklist for DSM-5 (PCL-5) is a 20-item self-reported measure that assesses PTSD symptoms experienced over the last month. Items assess symptoms across 4 symptom clusters of PTSD (re-experiencing, negative mood, avoidance, and hyperarousal) on a 0- to 4-point Likert scale. Total scores range from 0 to 80. A score of 33 or higher is associated with a likely diagnosis of PTSD [11].

Each mobile assessment consisted of 10 items. These included the 8 items (items 1, 4, 6, 7, 9, 12, and 18) of the abbreviated PCL-5 [8] and an additional item from the PCL-5 assessing sleep (PCL-5 item 20). The abbreviated PCL was used to minimize the burden to participants in that they had to complete 10 items as opposed to 21. The tenth item assessed pain on a scale of 0 to 10. Preliminary testing suggested it took approximately 5 min to complete each assessment. Each day for 30 days following initial assessment, the participants received a local notification on their mobile device to complete a survey. Participants had 10 hours to complete a survey regarding the symptoms for that day and were allowed to skip questions. Responses were uploaded immediately upon completion of each survey. After 30 days, participants received a notification that they no longer had to complete assessments but could continue to use the system for an additional 60 days at their discretion. Participants were compensated US $1 for each assessment completed within the first 30 days. The overall response rate for the combined sample was 78.0% (mean 23.33, SD 16.36 assessments). A majority of the sample (46/90, 51.1%) completed 75.0% or more of the assessments, resulting in 4312 assessments distributed over different days of the study. The rate of responding was compared with the mean rates reported in previous studies (mean 65.34%) [5,12,13]. Our study aimed to determine if elevated symptoms could be predicted solely based on these data collected via mobile phones as other input variables may not be available in certain clinical settings.

#### Data Selection

Data, in the form of 11 main features, were collected from each patient, namely, *Days.since.trauma*, *Reexp*_1_, *Reexp*_2_, *Avoid*_1_, *Avoid*_2_, *NACM*_1_, *NACM*_2_, *AAR*_1_, *AAR*_2_, *Sleep*, and *Pain,* shown and described in Table 1. To build the labels, a target variable was created for each row, Target_33_, based on the following conditions:

Target
_33_=1, when
*PTSD.Severity* ≥33.
Target
_33_=0, otherwise.

The experiments were conducted using a score of 33, which corresponds to a clinical cutoff for likely PTSD [11]. These cutoffs allow for the research to be conducted as a classification problem with a target value of either 0 or 1.

Although the features shown in Table 1 have been recommended by medical experts, in this study, we determined the feature relevance based on a given feature's ability to predict PTSD within the context of ML algorithms. The role of feature selection in this context is to ultimately reduce the number of symptoms that need to be assessed for accurate prediction. Proper feature selection should reduce overfitting and, therefore, increase accuracy as well as reduce model training and inference time [14].

#### Data Preprocessing

**Figure 1:**
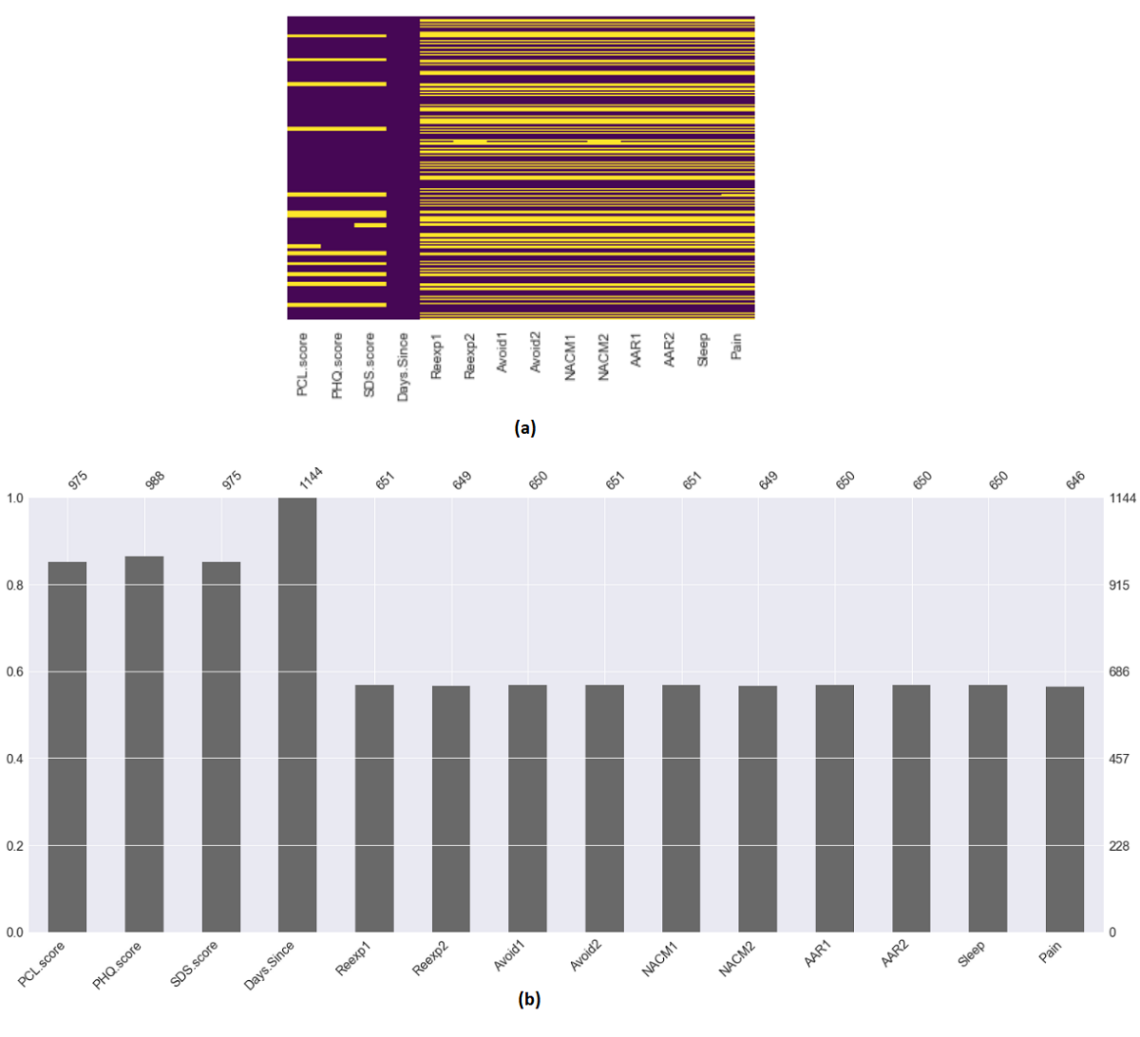
(a) Missing feature value distribution across all data input vectors. Yellow signifies missing values. (b) Missing feature percentage. *PTSD.Severity*: posttraumatic stress disorder symptoms. *Days.since.trauma*: days since trauma occurred. *Reexp*_1_: distress related to trauma-related intrusive thoughts; *Reexp*_2_: emotional reactivity to trauma cues; *Avoid*_1_: avoidance of thoughts about trauma; *Avoid*_2_: avoidance of environmental trauma-related reminders; *NACM*_1_: negative beliefs about self and the world; *NACM*_2_: loss of interest in activities; *AAR*_1_: exaggerated startle reaction; *AAR*_2_: difficulty in concentrating; *Sleep*: sleep difficulty; *Pain*: self-reported pain.

After determining the relevant features and target binary classification labels (PTSD or no PTSD at threshold value 33), the resulting data still contained a nontrivial amount of missing data. Specific patient response instances with missing values were not removed as they could potentially retain relevant information. Instead, missing values were replaced with an average calculated from the associated patient’s previous entries. Figure 1 shows the missing value distribution for each feature.

Standardization or normalization of features is a common preprocessing step in ML, producing features centered around a zero mean with unit variance. Feature standardization is a requirement for gradient descent–based ML algorithms (such as SVMs and logistic regression) for faster convergence and better performance. The general method for calculating standardized features is:

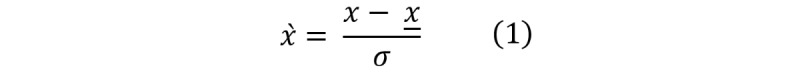



where for a given feature *x* is the original value, 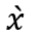
is the normalized value, 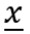
is the mean value, and σ is the SD.

**Table 1:** Dataset table (higher values signify more severe pathology).

Attribute (feature)	Description	Nonnull value	Range
*PTSD.Severity* (1 month)	Posttraumatic stress disorder symptoms 1-month posttrauma	975	0-80
*Days.since.trauma*	Days since trauma occurred	1144	1-49
*Reexp*_1_	Distress related to trauma-related intrusive thoughts	651	0-4
*Reexp*_2_	Emotional reactivity to trauma cues	649	0-4
*Avoid*_1_	Avoidance of thoughts about trauma	650	0-4
*Avoid*_2_	Avoidance of environmental trauma-related reminders	651	0-4
*NACM*_1_	Negative beliefs about self and the world	651	0-4
*NACM*_2_	Loss of interest in activities	649	0-4
*AAR*_1_	Exaggerated startle reaction	650	0-4
*AAR*_2_	Difficulty in concentrating	650	0-4
*Sleep*	Sleep difficulty	650	0-4
*Pain*	Self-reported pain	646	0-10

#### Feature Correlation

To identify both feature relevance and potential duplication of information surrounding early symptoms used to predict PTSD, we measured the correlation between all pairs of features. The correlation is statistically calculated between each feature variable and another using the average of the products between the standardized values of each sample. This process summarizes the relationship between features, known in statistics as the covariance method.

**Figure 2:**
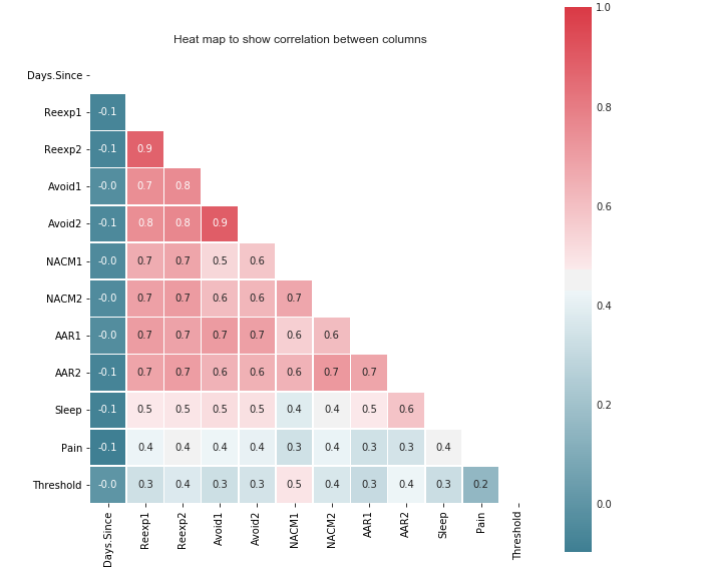
Correlation between features in our dataset, prior to feature selection. *PTSD.Severity*: posttraumatic stress disorder symptoms. *Days.since.trauma*: days since trauma occurred. *Reexp*_1_: distress related to trauma-related intrusive thoughts; *Reexp*_2_: emotional reactivity to trauma cues; *Avoid*_1_: avoidance of thoughts about trauma; *Avoid*_2_: avoidance of environmental trauma-related reminders; *NACM*_1_: negative beliefs about self and the world; *NACM*_2_: loss of interest in activities; *AAR*_1_: exaggerated startle reaction; *AAR*_2_: difficulty in concentrating; *Sleep*: sleep difficulty; *Pain*: self-reported pain.

In general, removing correlated features will not always enhance model performance but can aid in data preparation for ML algorithms. More importantly, this process can reduce the number of symptoms needed to predict PTSD. Figure 2 shows the correlation between features in our dataset. The aim of this correlation study was to reduce features in the event that 2 features are highly correlated. In particular, we noticed that *Reexp*_1_, *Reexp*_2_, *Avoid*_1_, and *Avoid*_2_ were highly correlated. Thus, *Reexp*_2_ was retained, whereas *Reexp*_1_, *Avoid*_1_, and *Avoid*_2_ were removed from our input feature set. Later, in the Results section, we discuss in detail the effect of this feature selection on model performance.

#### Feature Importance

**Figure 3:**
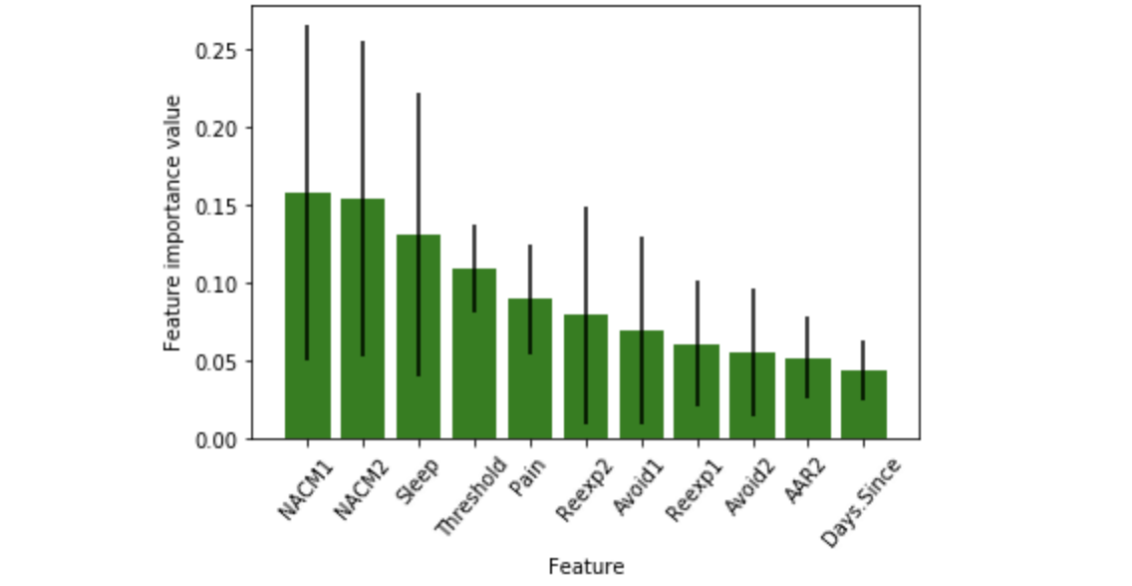
Ranked feature importance determined using the Gini method. *PTSD.Severity*: posttraumatic stress disorder symptoms. *Days.since.trauma*: days since trauma occurred. *Reexp*_1_: distress related to trauma-related intrusive thoughts; *Reexp*_2_: emotional reactivity to trauma cues; *Avoid*_1_: avoidance of thoughts about trauma; *Avoid*_2_: avoidance of environmental trauma-related reminders; *NACM*_1_: negative beliefs about self and the world; *NACM*_2_: loss of interest in activities; *AAR*_1_: exaggerated startle reaction; *AAR*_2_: difficulty in concentrating; *Sleep*: sleep difficulty; *Pain*: self-reported pain.

Feature importance methods score each feature by providing a quantitative measurement surrounding its relevance. The RF algorithm is capable of providing an importance score for each feature. RF can score the relevance of each feature through either statistical permutation tests or the Gini impurity index, which is used in this study, as shown in Figure 3. In the RF, a Gini impurity index is calculated at each node split using 1 feature variable to measure the quality of the split across classes at the considered node. The Gini impurity index is computed via the following equation:

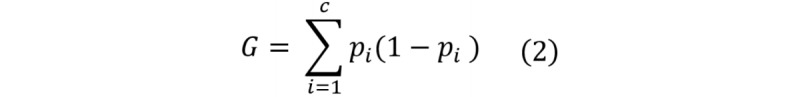



where *c* is the number of classes in the feature and *p_i_* is the fraction of samples labeled with class *i*.

To calculate feature importance, we sum the Gini impurity index values for each feature in the dataset over RF trees. These sums are then normalized and ranked to indicate the feature importance index. For more details on the Gini variable importance approach, see the study by Garcia-Lorenzo et al [15].

Features with smaller importance values can be removed from the dataset, thus, further reducing the number of relevant early symptoms to be used for PTSD prediction. Figure 3 shows that *AAR*_2_, *Avoid*_2_, and *Reexp*_1_ are less important than others. Furthermore, although the *Days.since.trauma* feature has a low score, this is an expected result, and this feature is, therefore, retained to provide important temporal information to the model.

In the Results section of this study, we discuss the effect of removing *AAR*_2_, *Avoid*_2_, and *Reexp*_1_ as they are low in importance, as well as *Reexp*_1_, *Avoid*_1_, and *Avoid*_2_, which are highly correlated with *Reexp*_2_, as discussed above. Notice that *Reexp*_1_ and *Avoid*_2_ are both low in importance and highly correlated with other features.

## Methods

In this paper, we studied multiple classifiers—logistic regression, naive Bayes, SVM, and RFs—to classify PTSD versus non-PTSD cases. In addition, we proposed *ensembles* of all these classifiers. It is known that ensembles of classifiers can form a better classifier than individual classifiers [16]. Ensemble methods combine predictions from several classifiers, or from a single classifier with different hyperparameters, to ultimately improve robustness as compared with a single estimator.

### Machine Learning Algorithms

Multiple binary classifiers were chosen for use in this study because of their established predictive power.

#### Logistic Regression

We applied logistic regression because it is widely used for binary classification problems. We built a linear classifier without performing any nonlinear transformation on the features. For more information about logistic regression classifiers, refer to the study by Held [17].

#### Naive Bayes

The naive Bayes classifier is simple, fast, and reliable and is derived from the Bayes theorem. The naive Bayes classifier assumes independent features with conditional independence, making the computation simpler (hence, *naive*). For more information about naive Bayes, refer to the study by Chan [18].

#### Support Vector Machines

SVMs are known for their generalization power, where the SVM kernel trick is used to implicitly enforce a nonlinear transformation on input features. In this study, we used linear, Gaussian Radial basis function (RBF), and polynomial kernels. We expect the results of the linear SVM to have similar or close results to the logistic regression classifier. For more information about the SVM algorithm, refer to the study by Burges [19].

#### Random Forests

RFs are an ensemble learning approach made up of multiple small decision trees, which are trained on a subset of data and features at each node split. In this study, we used RFs because of their predictive power and ability to work despite missing data (in light of missing data in our dataset). We did *not* replace missing data with associated average values for training RFs and, instead, we changed the relevant entries to be *−1*. For more information about RFs, refer to the study by Breiman [20].

#### Ensemble Methods

Ensemble methods work by combining several weaker classifier predictions, thus improving overall robustness. In this study, we ensembled the single classifiers: SVM (linear, Gaussian, and polynomial kernels), logistic regression, naive Bayes, and RF algorithms. We investigated 2 main techniques.

##### Hard Voting (Majority Voting)

In the case of hard voting, the final predicted class is taken to be the majority class label, as predicted by each individual classifier.

##### Weighted Average Probabilities (Soft Voting)

In the case of soft voting, the class label is calculated by summing the predicted probabilities across each class label and classifier and subsequently selecting the class with the highest probability. For this ensemble method, we used a uniform weight distribution.

### Prediction Performance Versus Days Posttrauma

To study the effect of time posttrauma on prediction performance, we trained the proposed classifiers on several different cutoff days. Specifically, we evaluated our models on data over 7, 10, 15, 20, 25, 30, 35, and all days posttrauma. Through comparative analysis, our aim was to determine how long surveys need to be performed to accurately predict elevated PTSD symptoms 1-month posttrauma.

#### Reducing Features

We also studied the effect of reducing the number of indicators (features) based on the feature correlation and feature importance methods discussed above. On the basis of these, we modeled without the features AAR2, Reexp1, Avoid1, and Avoid2, as they are highly correlated with other features or low in importance.

### Evaluation

Standard scoring metrics for ML models include accuracy (or error rate), true positive rate (TPR), false positive rate (FPR), true negative rate (TNR), false negative rate (FNR), recall-precision curves, and receiver operating characteristics (ROC) curves. These metrics provide a simple and effective way to measure the performance of a classifier [21]. In our evaluation, we focus on accuracy, confusion matrices, and ROC curves.

These scoring methods have been evaluated using 2 main methods: the holdout method [22] and the cross-validation method [23].

#### Holdout Method

For the implementation of the ML algorithms, our dataset was partitioned randomly into 70.0% and 30.0% for training and testing, respectively. The training set is used to train the models and to find the model hyperparameters, whereas the testing set is used to evaluate the model performance and its ability to generalize to new unseen data. The hyperparameters used for all the classifiers were manually assigned, and then hyperparameter tuning was performed using random search, as described in the study by Bergstra and Bengio [24].

#### Accuracy

Accuracy is a common metric to evaluate the performance of ML algorithms. It gives the ratio of correct predictions over the total number of predictions. In the case of imbalanced datasets, classification accuracy alone is insufficient to determine if the model is robust. For example, in a notable degenerative case, a model can predict only the majority class label and still achieve high classification accuracy.

#### K-Fold Cross-Validation

Models trained using a holdout technique might overfit or underfit depending on the distribution of the data split. To overcome this issue, K-fold cross-validation was performed on the dataset. This technique divides data into equal disjoint subsets of size K. The model being evaluated is then trained on all folds except one, which is reserved for testing. This process is then repeated K−1 times, selecting each fold to be used for testing one time. Finally, the results from each of the testing folds are averaged and returned as the final results. In this study, we used 10 folds, each fold is used once in testing and 9 times in training. This 10-fold cross-validation reduces the variance in the results by averaging over 10 different partitions, providing more reliable and generally accurate methodology than the Holdout method.

#### Confusion Matrix

Confusion matrices offer a comprehensive evaluation of the quality of an ML algorithm. In contrast to the singular dependence on 1 number from the accuracy metric, a confusion matrix provides a method of evaluating performance across all of the classes. For binary classification, the confusion matrix is simplified to 2 classes as follows:

[
*TP FP FN TN*]

where, TP is the number of true positives, FP is the number of false positives, FN is the number of false negatives, and TN is the number of true negatives. TP and TN represent the number of correctly predicted labels, whereas FP and FN are those that are mislabeled by the classifier. The higher true values in the confusion matrix the better, indicating more correct predictions.

#### Receiver Operating Characteristics Curve

The ROC curve is a simple graphical representation and powerful methodology to evaluate binary classifiers. It has become a popular method because of its ability to evaluate overall performance [25].

The ROC space is built and plotted using TPR and FPR from the equation TPR and FPR as the y-axis and x-axis, respectively. Each point (FPR, TPR) represents a classifier at a different threshold applied to the predicted labels’ probability [26] as shown by the following equations:

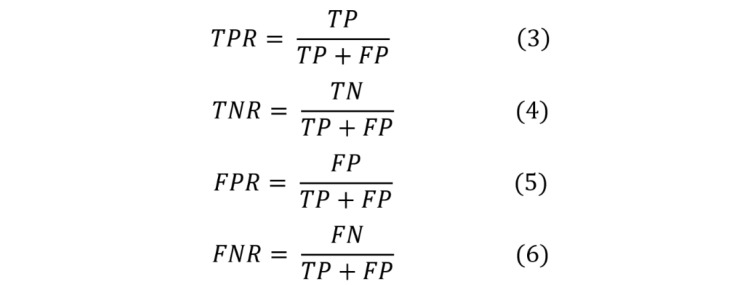



Independent of class distribution and error costs, the ROC curve connects the points in the ROC space. ROC curves describe the predictive performance and characteristics of a classifier at different probability levels. The area under the ROC curve, denoted as area under the curve (AUC), can be used to rank or compare the performance of classifiers [25]. AUC has been proven to be more powerful than accuracy in experimental comparisons of several popular learning algorithms [27], and in fact, we treat this as our *gold standard* evaluation method.

## Results

### Machine Learning Algorithms

For RFs, we used the Gini [28] algorithm to measure the quality of a split and 11 estimators. For logistic regression and SVMs, hyperparameter tuning was performed based on the random search technique described in the study by Bergstra and Bengio [24]. Results obtained after 50 random searches were as follows:

- Logistic regression, Lambda=0.02380
- SVM-linear kernel, C=62
- SVM-RBF kernel, C=57, Sigma =0.004
- SVM-polynomial kernel C=44, Sigma =0.017, degree=3

Where C and Lambda are the regularization terms and Sigma is the Gaussian kernel parameter. For ensemble methods, we investigated 2 main techniques: hard voting (majority voting) and weighted average probabilities (soft voting). For soft voting, we equally weighted the predicted probabilities from each classifier. We ensembled all the classifiers, that is, logistic regression, naive Bayes, SVM with linear kernel, SVM with Gaussian kernel, SVM-polynomial kernel, and RF.

### Accuracy

Table 2 shows the accuracy of various models in both the train-test split (holdout) and cross-validation methods. As shown in the table, the cross-validation can deal with the drawbacks of train-test split (holdout) technique, and therefore, it is a more reliable and generalized accuracy method than the holdout method. Thus, for the rest of our experiments, we exclusively used cross-validation.

### Receiver Operating Characteristics Curves

**Figure 4:**
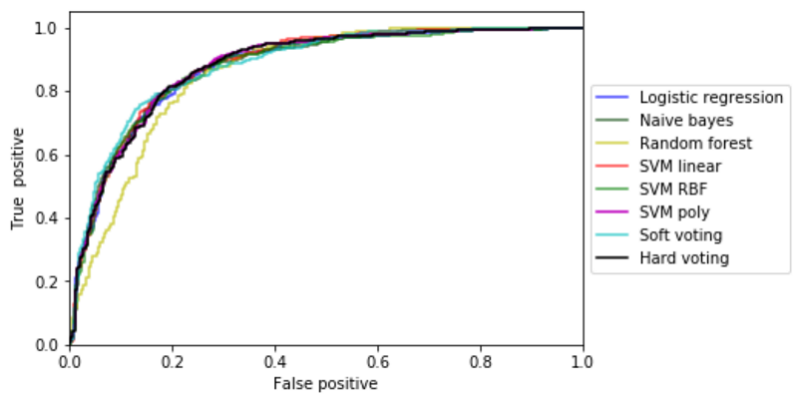
Receiver operating characteristics graphs for single and ensemble models. SVM: support vector machine; RBF: Radial basis function.

Figure 4 and Table 3 show the ROC curves for singular and ensemble models and the AUC.

### Reduced Features Analysis

**Figure 5:**
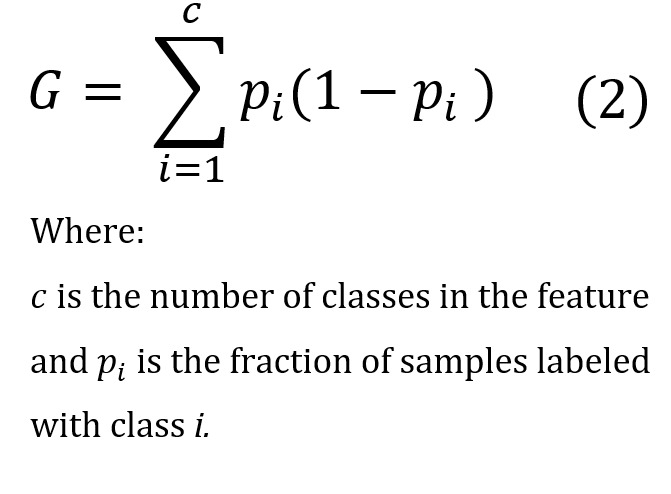
Receiver operating characteristics graphs for single and ensemble models with difficulty in concentrating, distress related to trauma-related intrusive thoughts, avoidance of thoughts about trauma, and avoidance of environmental trauma-related reminders features eliminated. SVM: support vector machine; RBF: Radial basis function.

We reduced the use of *AAR*_2_, *Reexp*_1_, *Avoid*_1_, and *Avoid*_2_ features because of their high correlation and low predictive power, as discussed in the Feature Correlation and Feature Importance dataset subsections. Figure 5 and Table 4 show the ROC curve and the AUC for singular and ensemble models, respectively, with those features eliminated.

**Table 2:** Accuracy results.

Machine learning method	Train-test accuracy	Cross-validation accuracy
Logistic regression	.8735236	.82110961
Naive Bayes	.8711414	.82210961
SVM^a^-linear kernel	.8349277	.76406263
SVM-Gaussian kernel	.8632561	.81908724
SVM-polynomial kernel	.8682245	.81857010
Random forest	.8212457	.77888143
Voting classifier-soft	.8798578	.82045190
Voting classifier-hard	.85919181	.80702013

^a^SVM: support vector machine.

**Table 3:** Area under the curve results.

Machine learning model	Receiver operating characteristics area under the curve
Logistic regression	.8325350
Naive Bayes	.8422145
SVM^a^-linear kernel	.8179543
SVM-Gaussian kernel	.8465576
SVM-polynomial kernel	.8337800
Random forest	.7844874
Voting classifier-soft	.8559346
Voting classifier-hard	.8357976

^a^SVM: support vector machine.

**Table 4:** Receiver operating characteristics area under the curve for reduced features models.

Machine learning model	Receiver operating characteristics area under the curve
Logistic regression	.87685409
Naive Bayes	.88154251
SVM^a^-linear kernel	.87553263
SVM-Radial basis function kernel	.88182758
SVM-polynomial kernel	.88158036
Random forest	.85092592
Voting classifier-soft	.88920514
Voting classifier-hard	.88145900

^a^SVM: support vector machine.

### Prediction Performance Versus Days Posttrauma

**Figure 6:**
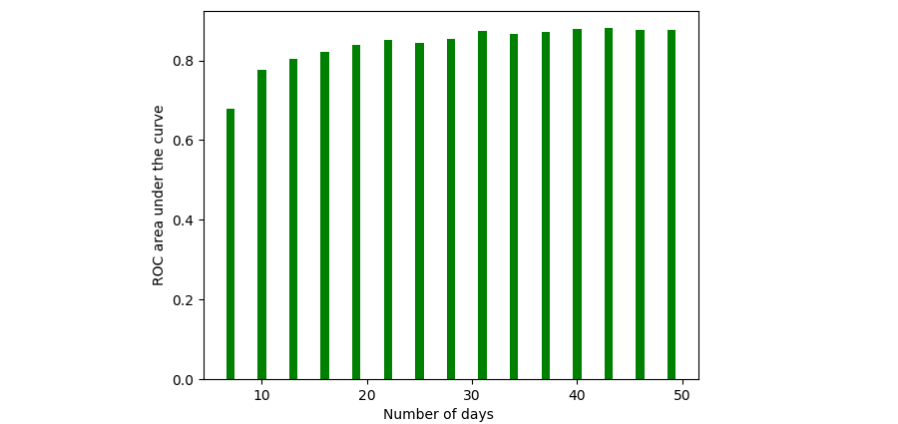
Area under the curve for the system trained and evaluated on different numbers of days. ROC: receiver operating characteristics.

Figure 6 shows the AUC of ROC curves for the ensemble model trained using the reduced features settings from the first 7, 10, 15, 20, 25, 30, and 35 days or all 45 days of patient data. These results demonstrate that an ensemble model has the same predictive power with 30 days of symptom reporting as it does with 45.

## Discussion

### Principal Findings

As discussed in the Results section, Figure 4 and Table 3 show that the SVM with a Gaussian kernel outperformed other single classifiers. SVMs usually generalize better than other ML algorithms as they maximize the margin between classes. It is also interesting to see that RFs performed comparatively poorly as it is a very powerful classifier and usually works well in case of missing data. Ensemble methods showed slightly better performance than single classifiers.

In addition, as shown in Figure 5 and Table 4, our results show significant performance enhancement by reducing features, indicating a high-variance system and suggesting that simplifying self-reporting questionnaires may yield better results. Reducing more features beyond *AAR*_2_, *Reexp*_1_, *Avoid*_1_, and *Avoid*_2_ did not improve the performance, indicating that these features might be considered noise and could be eliminated from the study. This reduction eliminated symptoms from the avoidance cluster of PTSD. Although these results suggest that the removal of these symptoms did not impact prediction, replication is needed before firm conclusions can be made about the role these symptoms play in PTSD prediction. Allowing for a shorter survey by removing these items reduces the burden of each assessment and is likely to increase survey compliance, which will provide a more accurate assessment of recovery.

Finally, as a key result, Figure 6 shows that the ensemble model can be used to predict elevated PTSD 1 month after a trauma, given that symptoms are displayed between 10 and 20 days posttrauma, with only a (5.0/100)% drop in performance. Each experiment in Figure 6 has been conducted independently. Thus, patients who are correctly classified using data from fewer days have no guarantee to be correctly classified by giving data from more days, even though it is very likely.

In summary, our results shed light on our research hypotheses stated in the Introduction section, as follows.

### Results for Hypothesis 1

An ML-induced ensemble model is able to demonstrate significant statistical correlations between observable symptoms and elevated PTSD 1 month after trauma with an AUC of 0.85, as shown in Table 3 and Figure 4. In addition, we have demonstrated that an SVM with Gaussian kernel outperformed other single ML algorithms.

### Results for Hypothesis 2

As detailed in the Results section, under the *Reduced Features Analysis* subsection, we have demonstrated that a subset of 7 standard early symptoms used to predict PTSD by care providers is adequate to predict elevated PTSD 1 month after a trauma.

### Results for Hypothesis 3

In the Results section, under the *Prediction Performance Versus Days After Posttrauma* subsection, we showed that an ensemble model has the same predictive power between 30 days and the full 45 days of the study period.

### Results for Hypothesis 4

In the Results section, under the *Prediction Performance Versus Days After Posttrauma* subsection, we showed how an ensemble model can be used to predict elevated PTSD 1 month after a trauma, given that symptoms are displayed between 10 and 20 days posttrauma, with only a (5.0/100)% drop in performance, as compared with a prediction at 30 days.

### Conclusions

Our experimental results are quite promising in that they suggest the potential for using a combination of self-reported symptoms and ML-induced models to automatically predict elevated PTSD in a manner that supports earlier interventions by care providers for 10 to 20 days posttrauma. These results were obtained using only data collected with a mobile device, suggesting that this method of symptom tracking is widely disseminable. Furthermore, our results suggest that smartphone surveys for self-reporting symptoms can be simplified more than previously understood.

We also explored various techniques for building predictive models. Although nonlinear learners did not outperform linear learners, an ensemble method with nonlinear models performed marginally better than single-linear models and will form the basis of our ongoing work in this area. In future studies, we plan to explore the application of these tools in a real clinical setting as a means to provide better care for at-risk patients. The prediction algorithm might also be improved if additional data were incorporated, such as baseline PTSD symptoms, demographic variables, and trauma histories, which is also an interesting topic for future studies.

## Abbreviations

AUC: area under the curve
FPR: false positive rate
ML: machine learning
PTSD: posttraumatic stress disorder
RBF: Radial basis function
RF: random forest
ROC: receiver operating characteristics
SVM: support vector machine
TPR: true positive rate
